# The interplay between recovery and immunity in infectious and non-communicable diseases

**DOI:** 10.3389/fcimb.2026.1745649

**Published:** 2026-07-15

**Authors:** Songlin Li, Ning Zhang, Xueting Jiang, Chunhong San, Xiaoxi Jiang, Ruiping Zhang

**Affiliations:** Panjin Liaohe Oilfield GEM Flower Hospital, Panjin, China

**Keywords:** immune modulation, immunity, infectious diseases, inflammation, recovery

## Abstract

**Background:**

The interplay between recovery and immune responses is increasingly recognized but remains under-integrated in clinical and research frameworks. Recovery extends beyond physiological repair to include restoration of function, psychological adaptation, and social reintegration, while immunity provides dynamic protection and regulation. Emerging evidence suggests that recovery trajectories shape immune competence and long-term resilience.

**Scope and approach:**

This review synthesizes evidence on bidirectional recovery–immunity interactions across infectious and non-communicable diseases (NCDs). We examine immune- linked mechanisms of inflammation resolution, tissue repair/regeneration, and functional restoration, and discuss how recovery states feedback to immune competence. Illustrative contexts include viral, bacterial, parasitic, and fungal infections, as well as cancer, autoimmune, metabolic, cardiovascular, and neurodegenerative disorders.

**Key findings:**

Shared pathways connecting recovery and immunity include cytokine signaling, macrophage polarization, and regenerative programs. Recovery outcomes are further modified by host and contextual factors such as genetics, aging, nutrition, comorbidities, and psychosocial stress. Clinical and translational approaches, such as immunotherapy, rehabilitation, vaccination, and precision medicine, offer practical ways to support recovery, but they must be applied with careful attention to the immune trade-offs they may introduce. Advances in biomarkers, multi- omics profiling, and AI-enabled modeling offer promising tools to quantify recovery phenotypes and predict immune resilience.

**Conclusion:**

Positioning recovery as an active immunological state enables more coherent frameworks for understanding disease trajectories and guiding interventions. Integrating recovery biology with immune regulation may enhance patient outcomes and support durable, resilient health across diverse disease settings.

## Graphical Abstract

**Figure:**
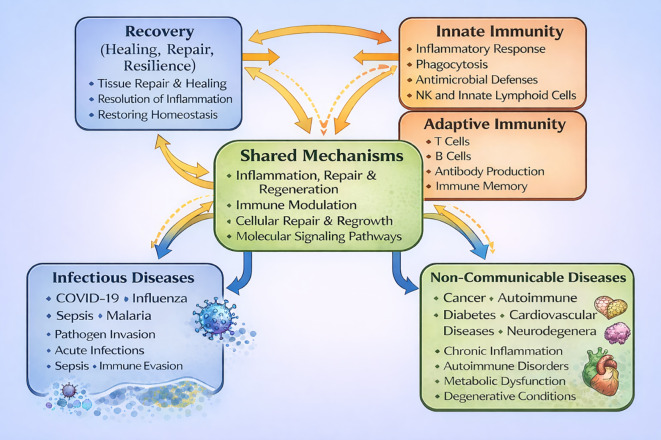
The interplay between recovery and immunity.

## 1 Introduction

### 1.1 Background and rationale

Both recovery and immunity are essential and interrelated processes indispensable to human health (Wahl et al., 2020). Recovery comprises the rebuilding of biophysiological roles (Staley et al., 2018), psychological comfort, and communal involvement after disease or injury (Adriani et al., 2018). In contrast, immunity defines the ability of the body to recognize, which has typically been overlooked and abridged, but it is gradually seen as crucial to illuminating the costs of both infectious and non-communicable diseases (NCDs (Noviello et al., 2015). COVID- 19 (Ashmawy et al., 2024; Ćwilichowska-Puślecka et al., 2025)), influenza (Lee and Kim, 2025; Sridhar et al., 2015), and sepsis (Prescott and Angus, 2018) are illustrations of infectious diseases that demonstrate the importance of advanced immune functioning for survival and recovery (Delano and Ward, 2016; Tosi et al., 2024). A clear illustration of disrupted recovery–immunity dynamics is provided by COVID- 19, where acute immune hyperactivation can resolve infection but leave prolonged immune dysregulation during recovery. Longitudinal studies have shown that many patients develop persistent inflammatory cytokine signatures, T-cell exhaustion, and autoantibody production, contributing to Long COVID and delayed functional recovery (Ashmawy et al., 2024; Ćwilichowska-Puślecka et al., 2025). This highlights how incomplete immune resolution can shape long-term recovery trajectories beyond the acute infectious phase.

Whereas NCDs, chronic diseases, like cancer and diabetes (Berbudi et al., 2020; Hu et al., 2024) and cardiovascular diseases (CVDs) (Girard and Vandiedonck, 2022; Golino et al., 2024), exemplify the insistent spiral of immune dysfunction that decelerates renewal of health and strength (X. Wang et al., 2025). Therefore, recognizing the interaction of these factors is perilous, surrounding the integrative process straddling from immunological sciences and translational medicine to rehabilitation disciplines (Augello et al., 2023; Lauterio et al., 2020; Wang et al., 2019).

### 1.2 Conceptual framework of recovery and immunity

To provide a consistent and translational perspective, the review is structured around a recovery-immune axis, which views recovery as an active, immune-regulated state. In this context, recovery is manifested as the organization of inflammation resolution, the restoration of immune homeostasis, and the process of tissue repair and functional regeneration. Immune programs determine the quality and duration of recovery through cytokine signaling, macrophage polarization, adaptive remodeling, and regenerative pathways, and recovery programs result in immune competence through metabolic and neuroendocrine control, psychosocial resilience, and immune memory. Each disease component is mapped to this model to show context-specific mechanistic differences, rather than general immune concepts that recur across infections, cancer, autoimmunity, metabolic and cardiovascular disease, neurodegeneration, and post-infectious syndromes, thereby enhancing clinical interpretability and intervention targeting.

**Figure 1:**
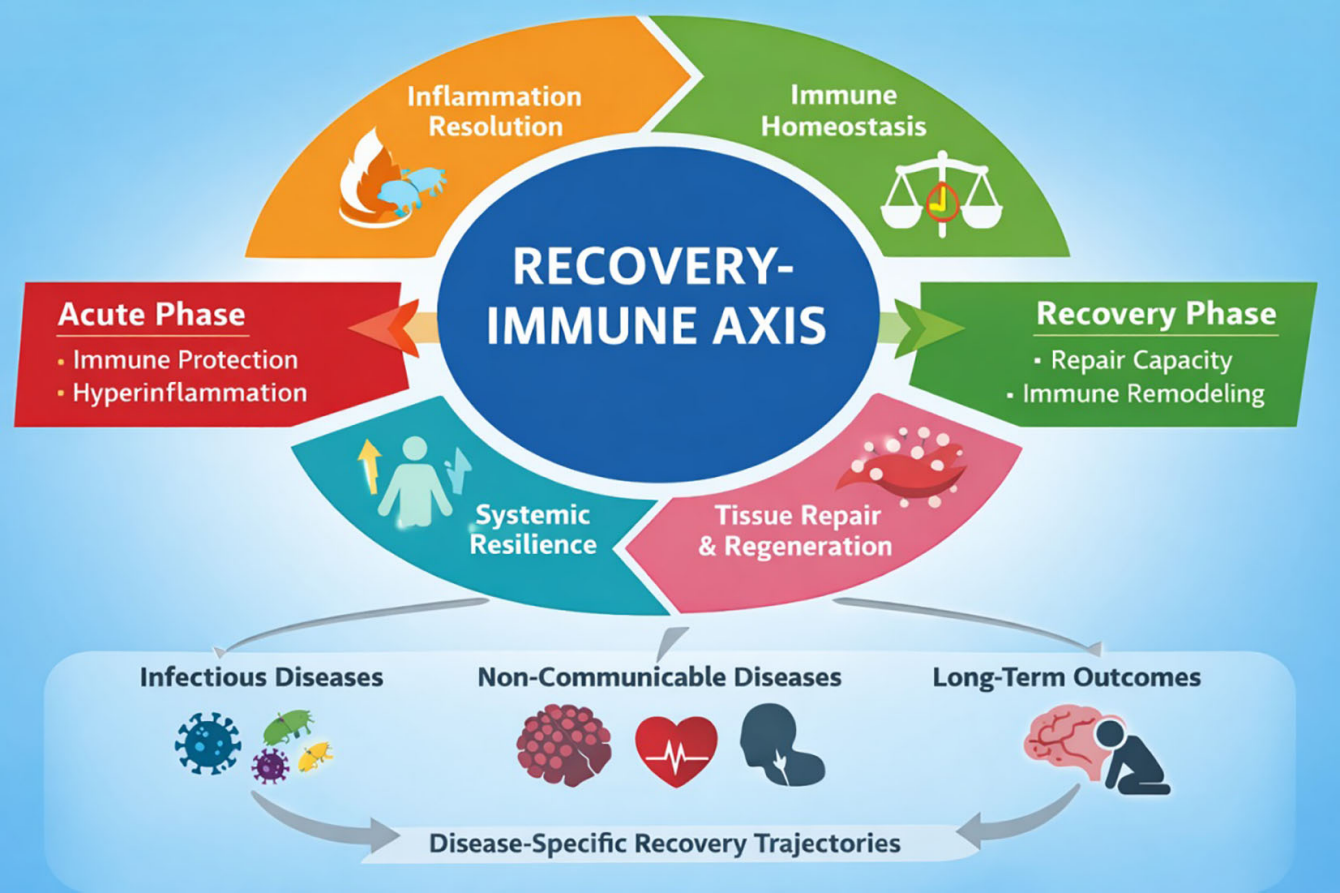
Schematic overview of the recovery–immune axis across disease categories. The figure illustrates shared immune pathways governing recovery, including inflammation resolution, immune homeostasis restoration, tissue regeneration, and systemic resilience. Disease-specific recovery trajectories are shaped by the balance between immune protection, repair capacity, and long-term immune remodeling.

As summarized in **Figure 1**, recovery is governed by coordinated inflammation resolution, immune homeostasis, tissue repair, and systemic resilience, with disease-specific trajectories shaped by immune remodeling. The conceptual framework of this review highlights the ever- changing and two-way relationship between recovery and immunity (Pandit et al., 2025). Active immune responses, on the one hand, facilitate pathogen clearance (Porte et al., 2019) and the resolution of inflammation and tissue repair (X. Wang et al., 2025). Recovery, on the other hand, interacts with and modifies immune competence by way of disordered metabolic reprogramming (Eichelmann et al., 2017), psychosocial and homeostatic resilience (Li et al., 2025; Noviello et al., 2015). The nature of this reciprocity interplay is context-dependent and heterogeneous with respect to disease, age, and the socio-environmental framework (X. Gao et al., 2025). From this perspective, the systems-level understanding of recovery and immunity increases the probability of finding new overlapping pathways and therapeutic targets, which are sorely needed in this time of unprecedented global public health challenges (Chiu, 2023).

### 1.3 Objectives and scope of the review

This review aims to:

1. Identify and explain both the similarities and the differences in recovery and immune response for infectious and non-communicable diseases.
2. Analyze molecular, cellular, and systemic levels of recovery and their immunological pathways.
3. Assess the psychosocial, genetic, and nutritional dimensions of these interrelations.
4. Analyze clinical and translational issues, including rehabilitative and precision medicine approaches, immunotherapy and immunological recovery.

The overall objective is to develop a model in healthcare which will be focused on quantifiable clinical and research domains and clinical ramifications of recovery-immunity dynamics.

## 2 Foundations of recovery and immunity

### 2.1 Definition and dimensions of recovery

As documented, recovery is multidimensional and symptomless, advocating for the well- being and functional ability restoration (Wahl et al., 2020). Physiological recovery implies balanced metabolism, restored organ functionality, and tissue repair (Zhang and Ioachimescu, 2024). Psychological recovery encompasses the realm, stress coping with illness burdens as well as revision of the illness-endorsed emotions and cognitions (Navarro-Ledesma et al., 2024). Functional recovery is the ability to return to the daily routines, work and social activities (Ralota et al., 2025). These aspects of recovery show that there is a level of health and quality of life that a person ought to attain and that recovery is not simply the absence of disease (X. Gao et al., 2025).

### 2.2 Overview of the immune system

Integrated defense and healing function of the human body are managed in two principal components of the immune system, that is, the innate and the adaptive immunity (Chi et al., 2024). Non-specific and immediate responses of the innate system are delivered through physical barriers and cytokines, and phagocytic cells (Batista et al., 2025; Rotshenker, 2011). Responses specific to the pathogen, generated by the crosstalk of T and B lymphocytes, reinforce immune responses and create memory storage for long-term protection and recovering from repetitive exposures (Chi et al., 2024; Enamorado et al., 2017; Guenin-Mace et al., 2023; Harrell et al., 2021). The immune system, involving T and B lymphocytes, ensures that inflammation is resolved, the area is cleared of any mycocytes and tissue inflammation, and mesenchymal tissue is repaired (Cressler and Adelman, 2024). Fibroblasts form new blood vessels in the region, enhancing tissue perfusion and aiding in recovery. If the regulation in either arm is disrupted, the body faces the risk of undergoing chronic inflammation, incomplete recovery, and key insults (Maddux et al., 2019).

### 2.3 Molecular and cellular mechanisms of recovery

**Figure 2:**
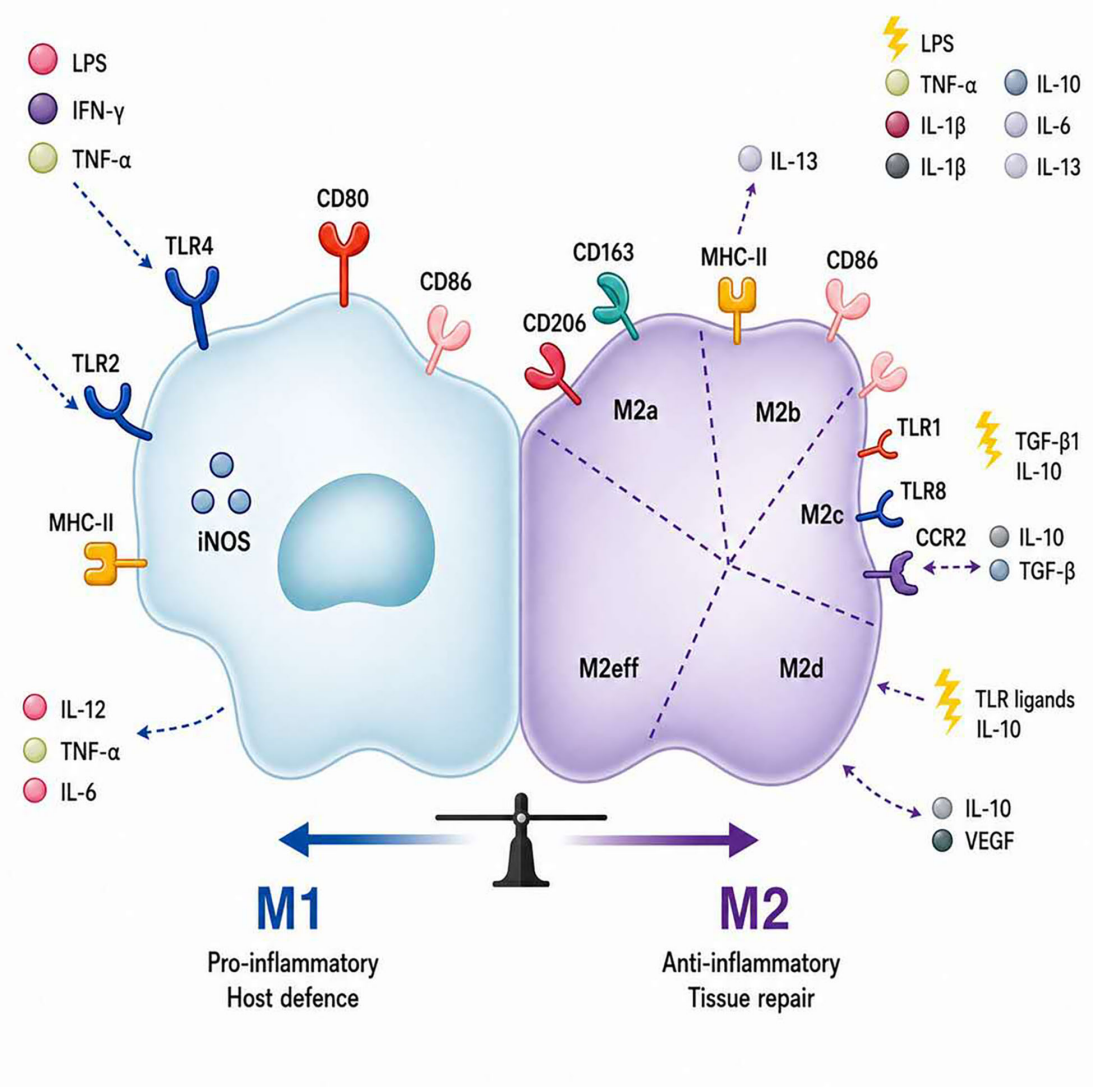
A presentation of the macrophage polarization in inflammation and tissue repair. While stimulated by LPS, TNF-α, or IFN-γ, M1 macrophages become highly pro-inflammatory, exhibiting markedly raised TLR-2, TLR-4, CD80, CD86, iNOS, and MHC-II expression. Cytokines such as IL-6, IL- 12, and TNF-α, released during the activation of the NF- κB and STAT1 signaling pathways, contribute to inflammation, enhancing pathogen elimination during the initial stages of wound healing. With the transition to the later stages of wound healing, macrophages embrace tissue repair, transitioning to anti-inflammatory, pro-repair M2 phenotypes. M2 macrophage subtypes have distinct functional profiles on M2 macrophage membranes. M2 macrophage subtypes and their characterized profiles include, M2a: IL-4 or IL- 13 activated macrophages that express CD206 and CD163 while producing IL- 10 and TGF-β, M2b: Immune complexes, LPS or IL- 1β trigger macrophages that express MHC-II and CD86 and secrete TNF-α, IL- 1, IL-6, and IL- 10, M2c: IL- 10 or TGF-β1 stimulate macrophages that express CD163, CCR2, TLR1, and TLR8 and have high levels of IL- 10 and TGF-β and TGF-β1. M2d: macrophages activated by TLR-4 or other TLR ligands and IL- 10, thus releasing IL- 10 and VEGF. M2eff: induced by efferocytosis and secretes TGF-β, PGE2, and PAF (W. Wang et al., 2025).

The cellular and molecular aspects of recovery are intricately governed by a fusion of immunological and non-immunological elements (Aiassa et al., 2023; Guenin-Mace et al., 2023). Of particular importance are the macrophages that give rise to and shift between inflammatory (M1) and repair (M2) phenotypes (Wynn and Vannella, 2016), immune regulatory T cells that participate in immune resolution (L. Wang et al., 2025), and downstream immune mediators (cytokines) such as the ones that promote angiogenesis, tissue repair and regeneration (**Figure 2**) (Caballero‐Sánchez et al., 2024; García-Martínez et al., 2025). There is established evidence that NF-kB, cytokine networks, immune cell metabolism, and cytokine reprogramming pathways are still critical to how inflammation and repair are integrated (Guenin-Mace et al., 2023; Li et al., 2025). Additionally, extracellular vesicles and microbiome-derived metabolites (Fidel Jr et al., 2020; Swiatczak and Rescigno, 2012) as well as epigenetically driven factors (Barreiro and Quintana-Murci, 2020; Zhang and Cao, 2019), are increasingly recognized as important constituents and modulators of recovery. Collectively, these mechanisms strengthen the integration of immune dynamics and recovery (Behura et al., 2023; R. Wang et al., 2025).

## 3 Interplay between recovery and immunity

### 3.1 Shared pathways of inflammation, repair, and regeneration

**Figure 3:**
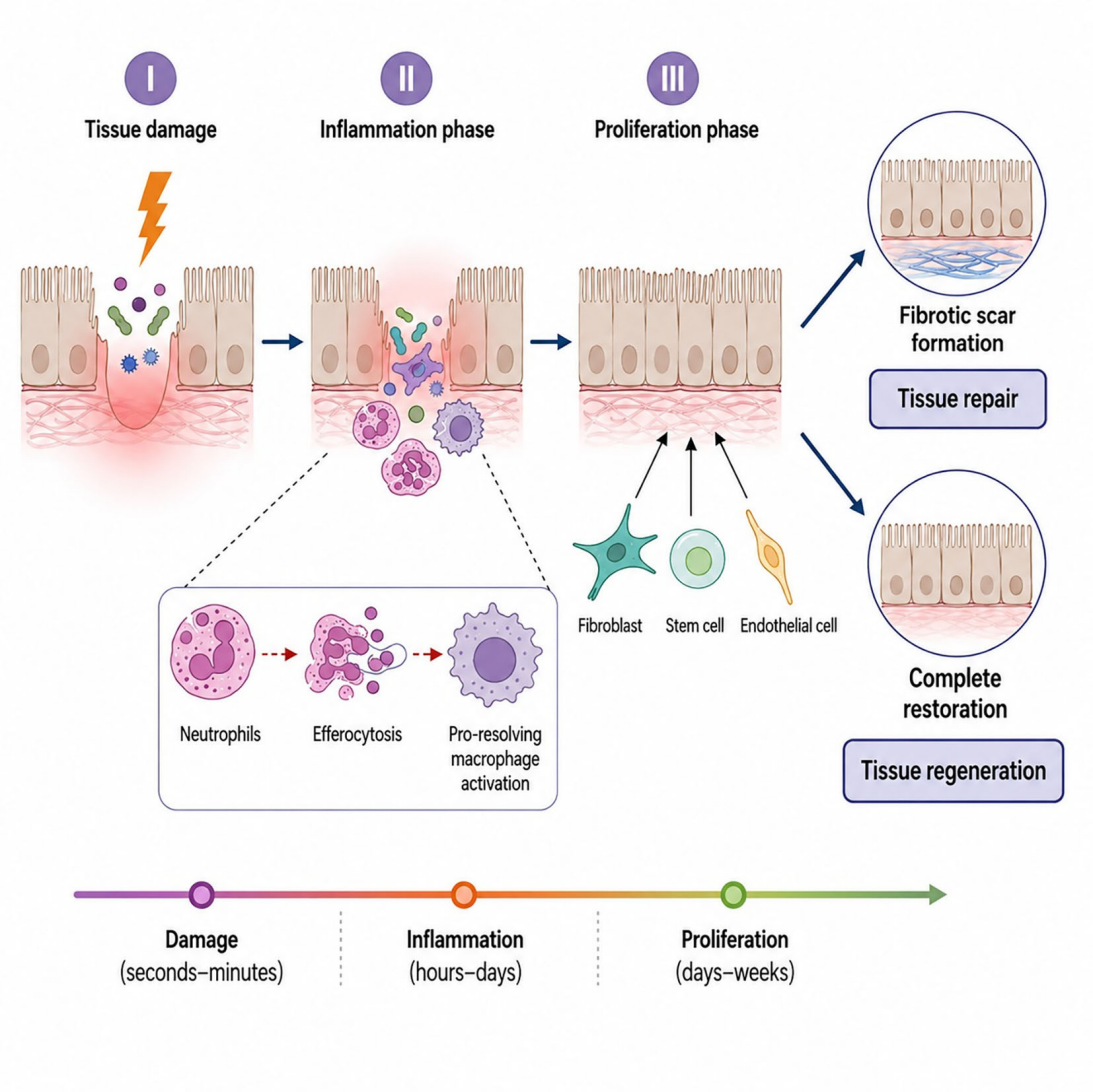
A summary of tissue repair and regeneration processes. The damaged tissue is the cause of the initiation of the inflammatory response when neutrophils reach the wounded site and secrete granular content to remove dead cells and pathogens. Neutrophils undergo apoptosis, engulfed by infiltrating macrophages through a mechanism called efferocytosis. Macrophages undergo a transition from pro-inflammatory to pro-resolving nature, resulting in the resolution of inflammation by secreting cytokines and lipid mediators. Once inflammation is halted, different stromal cells like fibroblasts, stem cells, endothelial cells, reach the wound site, forming granulation tissue, matrix and blood vessels and new tissue is formed. After this stage, the functional barrier of the tissue is restored, and scar formation occurs due to overactivation of fibroblasts and buildup of extracellular matrix. Regenerated tissue becomes a fully restored structure like that of the original one (Choi et al., 2023).

Like many biological processes, recovery and immunity interconnect in multiple ways. The intersections relate to the processes of inflammation, tissue repair and regeneration (**Table 1**, **Figure 3**) (Caballero‐Sánchez et al., 2024; Choi et al., 2023; Porte et al., 2019). During an immune response, the body’s primary goal is to eliminate pathogens (Zouali, 2025). This is done by heavily guarded and well-controlled interactions that occur in the body. Shared inflammation– repair pathways, including macrophage polarization and cytokine-mediated resolution (Section 2.3; **Figure 1**), provide a mechanistic foundation for recovery across diseases (Barrett and Puré, 2020; Oishi and Manabe, 2018). Regeneration, remaining undeterred, rejuvenates the tissue structure and function through stem and progenitor cells (Caballero‐Sánchez et al., 2024; Li et al., 2025). Failure in the steps of this continuum, in the case of inflammation, fibrosis, and scarring, results in looming consequences for self-defense and recovery processes, both of which rely heavily on one another (Choi et al., 2023; Wynn and Vannella, 2016; Zouali, 2025).

**Table 1:** A summary of the role of immune cells in the repair and regeneration of mammalian tissue (Choi et al., 2023).

Cell	Organ	Mechanism of action	Outcome
Macrophage	Skin	Lgr5+ stem cell activation by TNF-α, IL- 1β mediated expression of growth factors, Phagocytosis of Wnt inhibitors in scar formation	Regeneration
	Heart (Adult)	Collagen deposition at the wound site	Scarring
	Heart (Neonatal)	Angiogenesis at the infarcted site	Regeneration
Regulatory T cells	Hair follicle	Proliferation of follicle stem cells	Hair regeneration
	Intestine	Maintenance & proliferation of stem cells	Regeneration
	Brain	Secretion of osteopontin, polarization of tissue reparative microglia	Repair of white matter
γδ T cell	Skin	Synthesis ofFgf9	Hair follicle regeneration
	Bone	Synthesis of IL- 17A, Formation of osteoblasts from mesenchymal cells	Regeneration
ILC2	Intestine	Formation of amphiregulin and IL-22	Regeneration, Tissue protection
	Lung	Formation of amphiregulin	Regeneration
ILC3	Skin	Formation of IL- 17F	Repair
	Thymus	Proliferation of thymic epithelial cells by IL-22	Regeneration
	Intestine	Activation of the Hippo-YAP1 pathway in stem cells	Regeneration

### 3.2 Immune modulation in recovery processes

The pace and quality of recovery processes are determined and managed by the modulation of the immune system (Wahl et al., 2020). Pro-inflammatory cytokines (e.g., TNF-α, IL- 1β) must be counterbalanced by regulatory mediators such as IL- 10 and TGF-β to enable immune resolution and tissue repair (Tatsumi and Kumamoto, 2025). Immunomodulatory cells such as regulatory T cells, myeloid-derived suppressor cells, and alternatively activated macrophages accelerate an ultimate destructive immune response toward a destructive then reparative response (Chen et al., 2025). Corticosteroids, biologic therapies, and immunotherapies within the class of clinical medicine are designed and deployed to exploit this modulation, and hence accelerate immune recovery mechanisms within the context of both infectious and chronic diseases (Fallarino and Blank, 2025; J. Hu et al., 2025). Nonetheless, this indiscriminate immune recovery may induce immunosuppression and prolonged pathology, and therefore, making a balanced immunomodulation an essential component (Branchi et al., 2024).

### 3.3 Feedback loops: how recovery affects immune resilience

It is well documented that feedback loops shape future resilience and immune system recovery (Turner et al., 2023; Zhang et al., 2025). An effective repair of damaged tissue reinstates balance, diminishes chronic inflammatory signaling, and replaces energy reserves of immune cells (Chai et al., 2025; Wilson et al., 2023). Equally, incomplete recovery from organ damage and persistent inflammation weakens the immune system defenses and increases the susceptibility to recurrent infections and progressive disease (Chen et al., 2025). It is also reported that psychological and functional recovery affects immune resilience (Porte et al., 2019). Adequate rest and physical activity improve the immune responses of the body and enhance the adaptive immunity (Navarro-Ledesma et al., 2024), and excessive depression and fatigue weaken the immune system (Branchi et al., 2024). Recovery indeed modulates the system; immune activity follows recovery in ensuing health challenges. Reciprocal influences of the immune system and the brain are evident, as with frozen shoulder disease (Navarro-Ledesma et al., 2024) and infections after traumatic brain injury (Sharma et al., 2019). The involvement of the autonomic nervous system (ANS) influences the mechanisms of innate and adaptive immunity over the heart-brain axis. It also explains how CVDs influence cognitive roles and brain pathologies, leading towards cardiac problems (Simats et al., 2024).

An example of immune-recovery feedback in clinical settings is well established in post- sepsis syndrome, where survivors often experience prolonged immune suppression even after pathogens have been eliminated. According to Prescott and Angus (2018), sepsis recovery is often characterized by persistent immune exhaustion, increased susceptibility to secondary infections, and long-term functional dysregulation. These findings indicate that recovery is not only tissue repair but also the restoration of immune competence, which may take months or years to be complete (Delano and Ward, 2016; Prescott and Angus, 2018).

## 4 Recovery and immunity in infectious diseases

### 4.1 Viral infections

**Figure 4:**
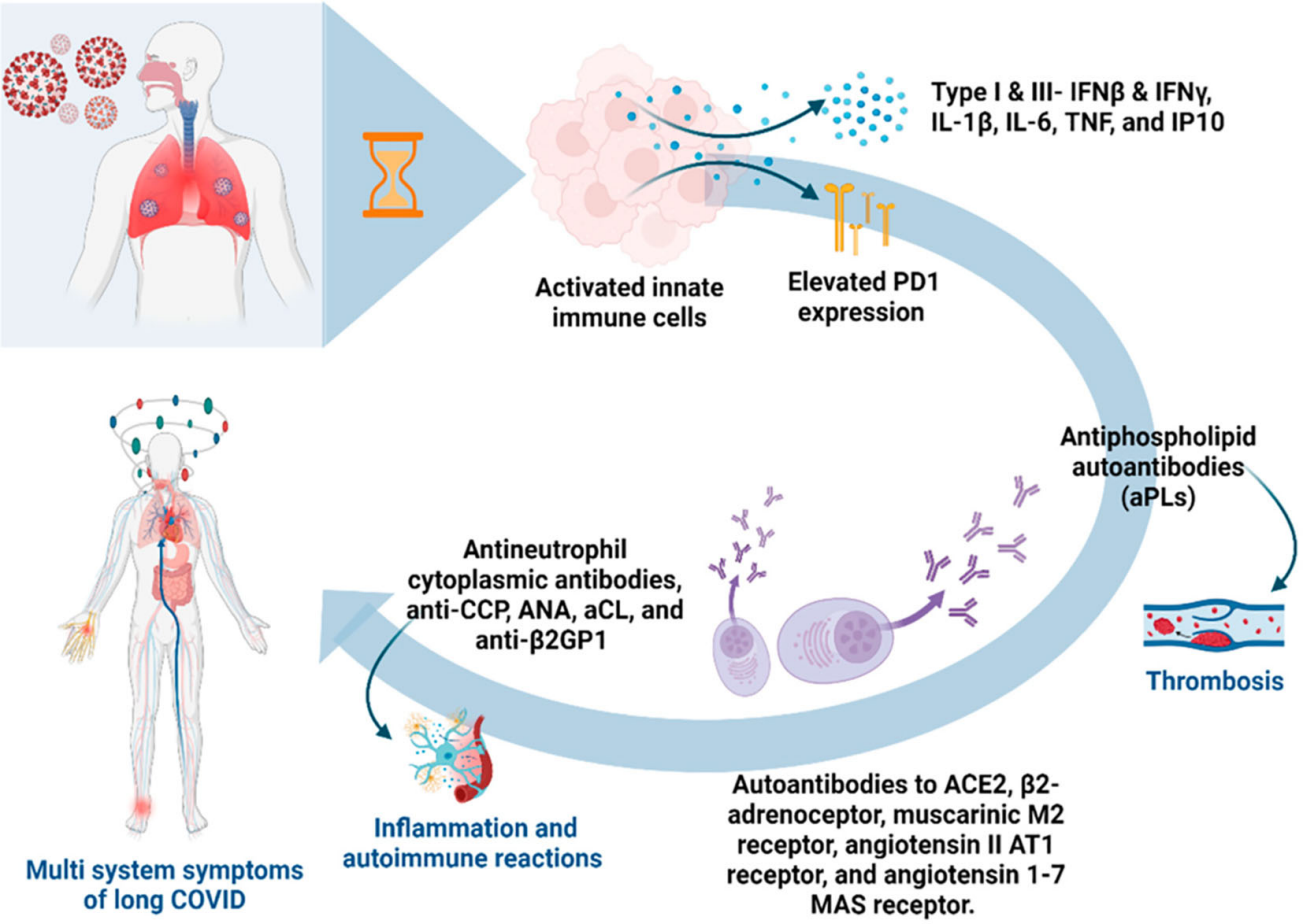
An overview of various types of immune responses and auto-antibodies present in long COVID patients. Reproduced from Ashmawy et al., 2024 Viruses 2025, 17(9), 1195, under the Creative Commons Attribution License (CC BY 4.0) (Ashmawy et al., 2024).

**Figure 5:**
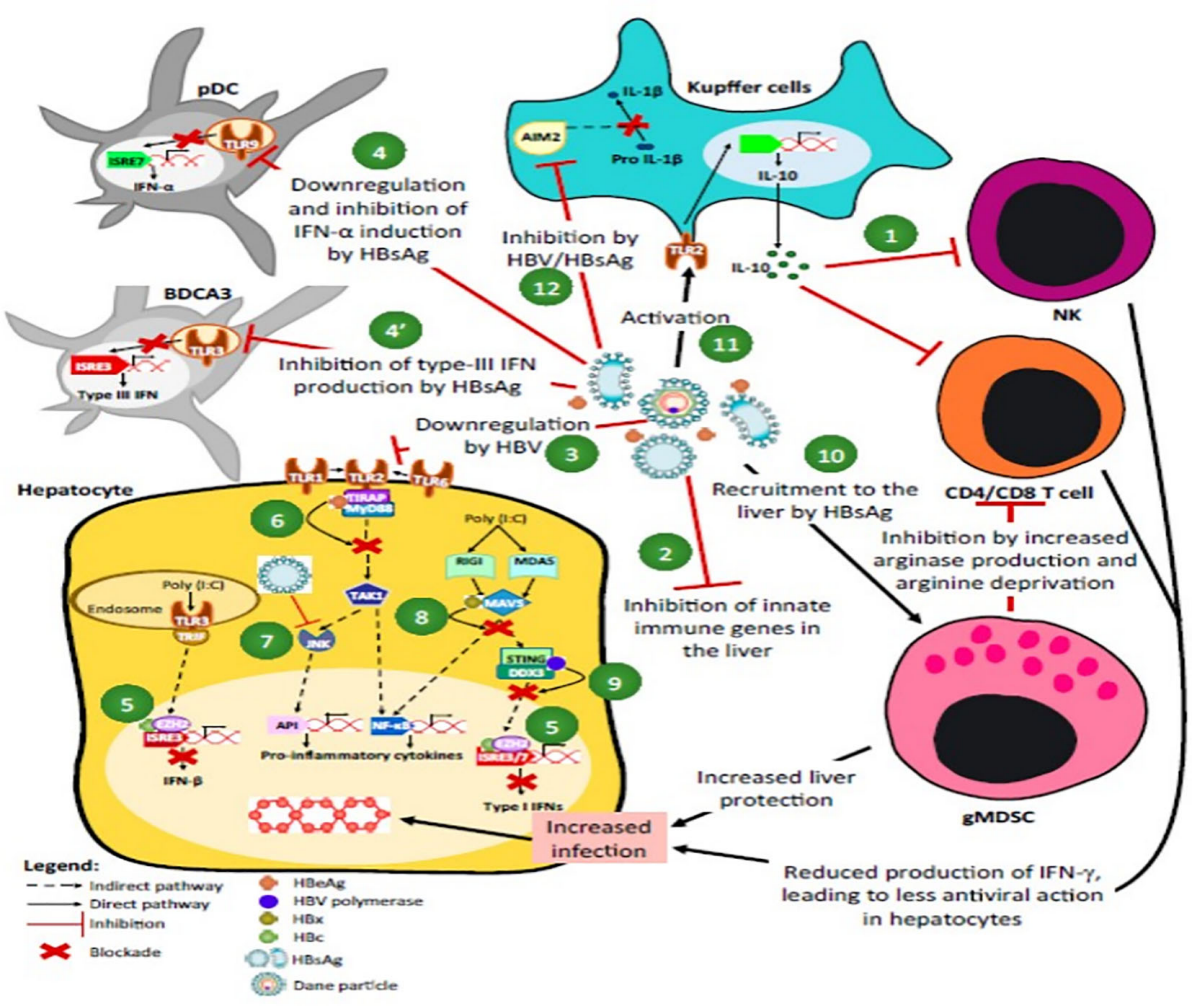
Modulation of innate immune sensors by HBV. (1) Interleukin‐10 production can diminish the NK cell activity and the non‐cytotoxic anti‐HBV action of IFN‐γ in infected hepatocytes. (2) HBV suppresses the induction of innate immune genes in the liver, and this is dependent on the expression of HBeAg and HBsAg. (3) HBV downregulates TLR2 in hepatocytes, which is associated with the decreased secretion of pro‐inflammatory cytokines/chemokines. (4) HBsAg downregulates TLR9 in plasmacytoid dendritic cells (pDC), resulting in the reduced secretion of IFN‐γ. (49) HBsAg decreases type-III IFN formation upon stimulation of TLR3, hence preventing a relevant activation of NK cells. (5) HBc can block the production of IFN upon stimulation of double-stranded RNA (dsRNA) recognition receptors by tethering the EZH2 complex and methylating immune promoters. (6) HBeAg binds to TIRAP/MyD88 complex to inhibit the TLR2 signaling pathway. (7) HBsAg blocks the JNK pathway, which prevents the production of IL- 12. (8) HBx silences the dsRNA-activated IFN response by associating with some host factors (MAVS, TRIF, IRF3) and/or inducing their proteasomal degradation. (9) HBV polymerase silences the dsRNA-activated IFN response by antagonizing STING and DDX3. (10) HBsAg can recruit and subsequently activate granulocytic myeloid-derived suppressive cells (gMDSC), which in turn, suppress the activity of CD4+ and CD8+ T cells by the expansive secretion of arginase, resulting in metabolic exhaustion. (11) HBV can activate Kupffer cells through the TLR2 pathway, resulting in IL- 10 generation. (12) HBsAg inhibits the AIM2-inflammasome, thus inhibiting the synthesis of IL- 1β, of antiviral effects on HBV replication in hepatocytes. Reprinted from Faure-Dupuy et al., 2017. Copyright 2017 by the authors. Licensee MDPI, Basel, Switzerland (Faure-Dupuy et al., 2017).

The interplay between immunity and recovery from illness has been illustrated by a variety of viral infections (Chen et al., 2023; Faure-Dupuy et al., 2017; Turk et al., 2024; Zarnitsyna et al., 2021). In the case of COVID- 19, immune hyperactivation results in acute pathology, but recovery depends on persistent inflammation of the lungs and restoration of pulmonary functioning (Ahmad et al., 2020; Batista et al., 2025; Kamińska et al., 2022; Lauterio et al., 2020). Long COVID exemplifies how disrupted recovery trajectories can stem from imperfect immune resolution (**Figure 4**) (Ashmawy et al., 2024; Ćwilichowska-Puślecka et al., 2025). Innate cells like NK cells, dendritic cells, and NKT cells in the liver are actively involved in chronic hepatitis B virus (HBV) pathogenesis, as well as therapeutic failure (**Figure 5**) (Faure-Dupuy et al., 2017; Yang et al., 2022; Zheng et al., 2023). In the case of influenza, immune memory helps speedy recovery from reinfection, though the outcomes may become more complicated by secondary bacterial infections (Chen et al., 2023; Fan et al., 2025; Sridhar et al., 2015).

The depletion of CD4+ T cells and continuous inflammation in HIV profoundly impedes recovery. It indeed illustrates how the viral core persistent shifts the pivotal immunological and restoration balance (Chen et al., 2023; Fidel Jr et al., 2020). Resting T cells and repairing T cell exhaustion, in part, through epigenetics, is being studied within the framework of IL-7 therapy (Augello et al., 2023; Berendam et al., 2022; Obeagu, 2025). Along with DNA methylation, epigenetic immune regulatory genes, histones, and HIV non-coding RNA profoundly modulate replication of HIV particles and immune phenomena in HIV positive individuals (Fidel Jr et al., 2020; Obeagu, 2025). These points illustrate how advanced recovery correlates with the dynamics of suppression and control of immune antiviral response (**Table 2**) (Batista et al., 2025; Berendam et al., 2022; Fidel Jr et al., 2020; Zhang and Cao, 2019).

**Table 2:** Impact of SARS-CoV-2 proteins on NK cell dysfunction and cytokine signaling (Batista et al., 2025).

ViralProtein	Effect on NK cells	Impact on cytokines	Consequences
Spike	**↓** MICA/B (inhibits stimulation)	**↑** IL-6, **↓** IFN-γ	Phenotypic collapse of NK cells
ORF3a	**↑** PD- 1/TIM-3 (exhaustion)	**↑** TGF-β (Immunosuppression)	Loss of cytotoxic role of NK cells
NSP1	**↓** CD69 (stimulation)	Change of type I IFN pathways	Weak antiviral response due to reduced immune cell stimulation

↑, Increase; ↓, decrease.

### 4.2 Bacterial infections and sepsis recovery

In the case of bacterial infections, the most difficult aspects are the tissue damage inflicted by the pathogen and the host, and the counter-immune responses (Fan et al., 2025) (**Table 3**). In sepsis, uncontrolled initiation of the immune system is linked with relentless inflammation, failure of multiple organ systems, and a very high death rate (Tosi et al., 2024). Most patients who survive enter a prolonged recovery phase characterized by immune system exhaustion, high vulnerability to secondary infections, and significant long-term functional disabilities (Delano and Ward, 2016; Prescott and Angus, 2018). Recovery is achieved through mechanisms of immune reshaping, clearance of the microbial insult, and tissue repair, but most patients addressing the remaining aspects of post-sepsis syndrome complete a recovery that is often incomplete (Tosi et al., 2024). Recovery achieved with purely rehabilitative maneuvers (Ahn et al., 2018; Ferreira, 2025), and recovery of the physiological function and immune system after targeted nutrition (Nieman and Mitmesser, 2017) is highly encouraging. The leading site of bone marrow is colonized by the memory CD4+ T cells of patients during the recovery from sepsis (Skirecki et al., 2020), and it has been reported that the recovery from sepsis is accompanied by long-standing impairments in the CD4+ T cell responses (Ammer-Herrmenau et al., 2019; Turk et al., 2024).

**Table 3:** Summary of immune modulators with sepsis therapy benefits (Delano and Ward, 2016; Marques et al., 2023).

Immune modulators	Properties	Implied benefits	Key references
G-CSF (Granulocyte CSF)	A glycoprotein that induces the synthesis of granulocytes	Increases the production and release of granulocytes and macrophages. Enhances myelopoiesis, granulopoiesis and monocyte production	(Lieschke et al., 1994; Nelson et al., 1998; RK, 2003; Winters et al., 2010)
GM-CSF (Granulocyte- macrophage CSF)	Augments monocyte and lymphocyte functions	Improves neutrophil and monocyte formation and function; enhances monocyte and lymphocyte cytotoxicity; improves monocyte and lymphocyte provocation, ballistic T cell augmentation, and nosocomial infections	(Boomer et al., 2011; Francisco-Cruz et al., 2014; Hall et al., 2011; Meisel et al., 2009; Paine III et al., 2012)
IFN-γ	Crucial in viral, bacterial and protozoal infections, a key inducer of macrophages for the MHC-I complex	Augments monocyte functions and HLA-DR molecules, anti-infection functioning, and improvements in the complications of fungal infections	(Cuenca et al., 2013; Döcke et al., 1997; Dries et al., 1994; Nalos et al., 2012; Xiao et al., 2011)
PD- 1/PD-L1 (Programmed	Expression in T and B lymphocytes	Potential biomarkers for candidates for immune modulating therapy;	(Chen and Flies, 2013; Day et al., 2006;
cell death protein 1 & ligand)	and concurrent myeloid cells, sending inhibitory signals to reduce PD-L1 and CD+ to T cell over- and rest in the epithelium. PD-L1 is expressed to a high degree on CD4+ and CD8+ T cells during viral infection and neoplasia.	Reverse T cell exhaustion; Stimulate lymphocyte propagation; Enhance neutrophilic and monocytic cell- mediated cytotoxicity; Control opportunistic infections	Topalian et al., 2012; Zhang et al., 2011)
IL-3	Interleukin-3	Augments the propagation and recruitment of T cells; Reduces the apoptosis of lymphocytes; Enhances the synthesis of IFN-γ from T cells; Improves T cell homing and clearance of pathogens; Enhances the diversity of T cell receptors	(Hotchkiss and Sherwood, 2015; Weber et al., 2015)
IL-7	Interleukin-7	Augments the proliferation and recruitment of T cells; Reduces the apoptosis of lymphocytes; Enhances the secretion of IFN-γ from T cells; Improves T cell homing and clearance of pathogens; Enhances the diversity of T cell receptors	(Demaret et al., 2015; Kinter et al., 2008; Mackall et al., 2011; Perales et al., 2012; Unsinger et al., 2010)
IL- 15	Interleukin-15	Lowers the rate of apoptosis of NK cells, T cells, and NKT cells; Enhances their production and activation	(Inoue et al., 2010; Pelletier et al., 2002).

### 4.3 Parasitic and fungal infections

Cases of parasitic and fungal infections typically comprise long-lasting engagements of host defense and the pathogen’s evasion tactics (J. Liu et al., 2024; Sais et al., 2024). Recovery from parasitic diseases such as malaria and leishmaniasis involves a delicate equilibrium between the destruction of the parasite and mitigating the damage to the host tissues stemming from uncontrolled inflammation (Dobbs et al., 2022; Florini et al., 2024; Shelton et al., 2015; Volpedo et al., 2021). It enabled the determination of rapidly altered pathways and provided additional insight into the preliminary molecular cross-talks associated with Leishmania and the host cells (Atri et al., 2025). The converse correlation of TLR1/2-TGF-β is central to the tissue- permissive parasite persistence during Leishmania infection (Roy et al., 2025). Fungal infections, such as aspergillosis or candidiasis, exemplify the role of innate immune cells like neutrophils, macrophages in the early stages of defense, followed by the adaptive responses needed for clearance and recovery (Chen and Gao, 2024; Gibert et al., 2025; B. Liu et al., 2024). Immunocompromised patients usually fail to recover completely due to the lack of antifungal immune response, which situates immune competence as a crucial factor in recovery pathways (Chiusaroli et al., 2025; Frederick et al., 2025).

### 4.4 Long-term immunity and post-infectious syndromes

In some cases, recovery from an infection is incomplete, and many people suffer from post-infectious syndromes (Abhimanyu et al., 2021; Bannister, 1988). Post-fatigue syndromes, chronic lung dysfunction following pneumonia, and neurological sequelae are examples of incomplete recovery with lingering immune changes (Cherepakhina et al., 2009; Molina-Holgado and Molina-Holgado, 2010; Ruiz-Pozo et al., 2025; Stormorken et al., 2015). On the other hand, the immune memory developed as a result of recovery offers a form of long-term protection, as seen with adaptive immunity after recovery from or vaccination against measles, varicella, or other viruses (Li et al., 2025; Rios et al., 2025). The durability of immune memory, however, remains dependent on the specific pathogen, host characteristics, and the chance of reinfection or relapse (Cressler and Adelman, 2024; Lei, 2025). The enduring imbalance of post- infectious complications and the immunity thereof remains an area of focus for developing and fine-tuning therapeutic prevention (Abhimanyu et al., 2021; Alsaadawe et al., 2025).

A significant case of impaired recovery-immunity integration is given by long COVID and other post-infectious syndromes, where clinical recovery is not always concomitant with immune resolution. Although acute SARS-CoV-2 infection can resolve, a substantial proportion of patients exhibit persistent immune dysregulation in convalescence, including persistent inflammatory cytokine signatures, T-cell exhaustion, and autoantibody formation. Such abnormalities may lead to long-term symptoms, delayed functional recovery, and chronic post- acute consequences (Ashmawy et al., 2024; Ćwilichowska-Puślecka et al., 2025). These post- infectious recovery syndromes indicate that immune activation, though necessary for pathogen control, can also entail long-term trade-offs during recovery when inflammation does not fully resolve.

The IL-27, which is an anti-viral, pro-inflammatory, and anti-inflammatory cytokine secreted by dendritic cells, macrophages, and B cells, during an immune response, has been documented in the regulation of IL-27 (Andres-Martin et al., 2024). These IL-2 family cytokines, IL-21 and IL-9, also demonstrate differential regulation of innate and type 2 immunity in asthma (Bick et al., 2024). A role for IL- 18 cytokine has been proposed in regulating the migration of macrophages and monocytes during the recovery from surgery. The ratio of IL- 18/TNF-α as a new marker for the resolution of inflammation after surgical intervention has been proposed (Papatheodorou et al., 2025). An imbalanced change of cytokine expression among T helper 1 and T helper 2 (Th1/Th2) cells in the intestine of the post-infectious IBD (Chen et al., 2012). Recent literature has described that the post-infectious IBD is accelerated by an increased level of adenosine 2A receptor, which promotes T polarization of CD4+ T cells to Th17 (Dong et al., 2022). Trying to treat this condition, atractylenolide I (AT-I) has been shown to improve post- infectious IBD by blocking the polymerase I and trx release factor and the c-Jun N-terminal kinase/inducible nitric oxide synthase (NOS) pathway (Jianan et al., 2025). More studies on the efficacy and safety of microbial-targeted therapy in patients with this disease are needed (Lupu et al., 2023; Sundin et al., 2015).

## 5 Recovery and immunity in non-communicable diseases

### 5.1 Cancer: immune surveillance and post-treatment recovery

Cancer serves as a prime example of the relation between recovery and the immune system (Fang et al., 2025). During the recovery period, the immune system is fundamental in immune surveillance: spotting and eliminating cancerous cells (J. Hu et al., 2025). Nevertheless, tumor cells hide from the immune system by ways of immune checkpoint expression (Li et al., 2025) through the use of suppressive microenvironments. Cancer therapies can impair immune competence, increasing susceptibility to infection and delaying recovery (Koti et al., 2023). One of the most significant case studies of recovery-immunity integration is the clinical application of CAR-T cell therapy, which has the potential not only to eliminate malignant cells but also to redefine immune recovery following extensive cancer treatment. Jiang et al. (2022) demonstrated that the use of granulocyte-macrophage colony-stimulating factor (GM-CSF) increased CAR-T cell proliferation and accelerated immune recovery without cytokine release syndrome (Jiang et al., 2022). It illustrates how robust immune reconstruction and regulated immunomodulation are crucial to oncology recovery outcomes. On the other hand, immunotherapy, which includes checkpoint inhibitors and CAR-T (chimeric antigen receptor T-cells), describes how recovery from cancer can be aided by recovery from cancer immune therapeutics (Fang et al., 2025; Jiang et al., 2022). Cancer survivorship should incorporate immune system recovery strategies along with the rest of the body’s system, as well as from psychosomatic care (Wang et al., 2019).

### 5.2 Autoimmune disorders: systemic dysfunction of the immune system

Rheumatoid arthritis (RA), alongside other autoimmune diseases, multiple sclerosis (MS) (Fominykh et al., 2023; Maciak et al., 2025), IBD (Doroszkiewicz et al., 2024; Goethel et al., 2018), and systemic lupus erythematosus (SLE) (Lindblom et al., 2025) serve to illustrate the disruption to recovery caused by the dysfunctional immune system. In this instance, the immune system is literally autoimmune. It targets the tissues of the host while simultaneously and profoundly hindering reparation systems (Maciak et al., 2025). Recovery is left with the complexity of chronic inflammation, scar tissue, and indefatigable disease activity (Lee and Kim, 2025). Therapies address the fundamentals of unsystematically organized immune reaction while permitting the requisite immune competence for repair and protective (Tabatabaei et al., 2025). Yet, sustained treatment through immunosuppressants may limit complete recovery as they increase susceptibility to infections and metabolic derangements (Mancuso et al., 2022). This embodies the challenge of restoring the balance between immune control and regenerative recovery (Caballero-Sánchez et al., 2024). Certain gut microbiota abnormalities (Gao et al., 2024) with reduced diversity and increased pro-inflammatory organisms may contribute to the obesity- related autoimmune disorder immunology of SLE and suggest new avenues for therapy (Pillai et al., 2025).

### 5.3 Metabolic disorders

**Figure 6:**
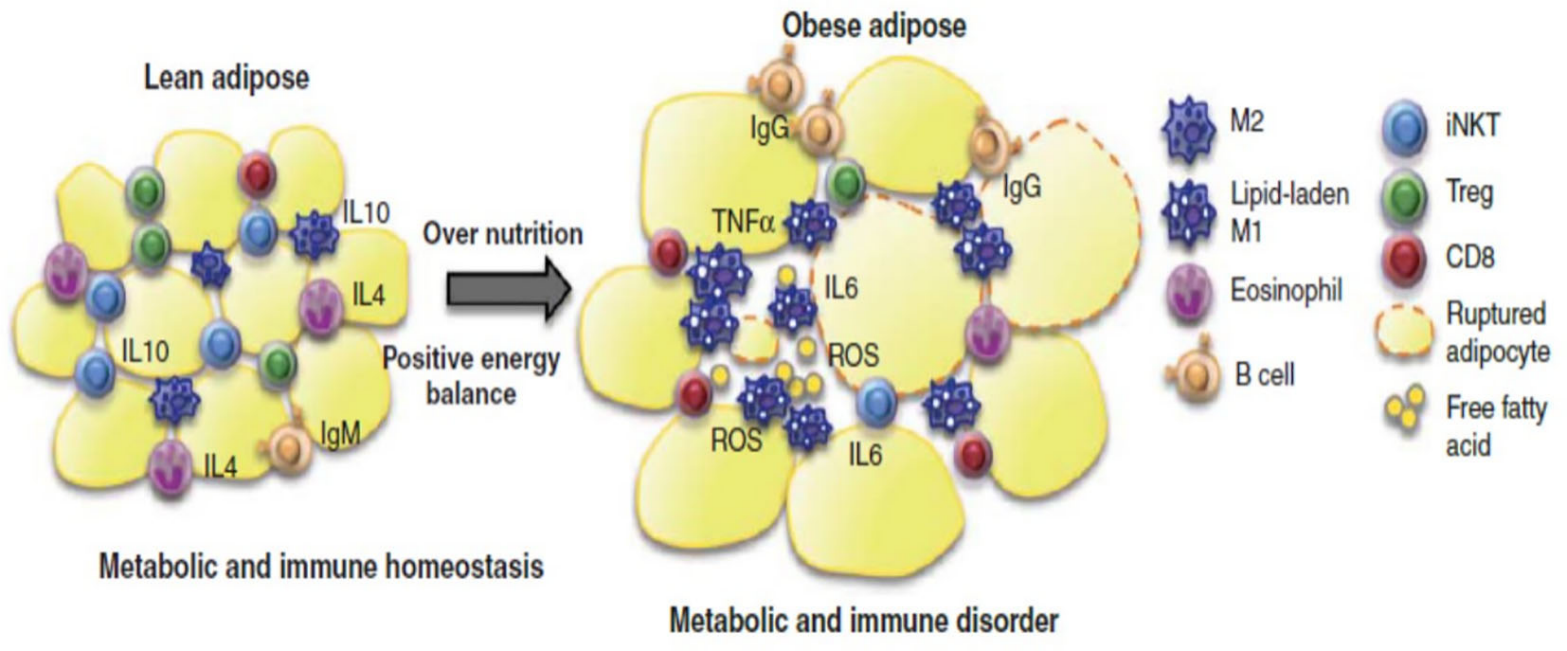
Adipose homeostasis during steady-state and obese conditions. In a lean condition, immune cells and adipocytes stay in proximity and engage in a collaborative effort toward homeostatic and constraining lipid handling and storage by adipocytes. Among the primary immune cell residents are the iNKT cells, Tregs, eosinophils, IgM B cells, and M2 macrophages, which are alternatively activated. iNKT and Tregs, and M2 macrophages producing IL- 10 and eosinophils producing IL-4 help to preserve a tolerogenic atmosphere. In the expansion phase of obesity and excess adipose tissue, iNKT cells and Tregs are absent, and a shift of macrophage phenotype from M2 to M1 occurs that accumulates around overloaded and rupturing adipocytes. Reprinted from Mark et al., 2014. Copyright 2014 Society for Endocrinology Printed in Great Britain (Exley et al., 2014; Wang et al., 2024).

Immune malfunction has been noted to significantly contribute to difficulties in recovering from ailments, including diabetes and obesity (Berbudi et al., 2020; Collins et al., 2024; Exley et al., 2014; Holder and Reddy, 2021). The tissue repair process and the metabolic system equilibrium get disrupted, the repair process is slowed down, and the chronic low-grade inflammation is on account of adipose tissue macrophages and specified cytokines (L. Wang et al., 2025; Yang et al., 2015). Patients suffering from diabetes have low-functioning immune cells, wounded and damaged vessels, and oxidatively stressed, which causes the patient to take time healing wounds with increased susceptibility (Berbudi et al., 2020; Doroszkiewicz et al., 2024; Hu et al., 2024). Exacerbated immune responses, in turn, cause and promote insulin resistance (Wang et al., 2024). Hence, the recovery for these patients is more complex, which may include metabolic intervention and immune control, in terms of lifestyle changes, integrated approaches, and nutritionally focused therapy (**Figure 6**) (Holder and Reddy, 2021; Huo et al., 2025; Nieman and Mitmesser, 2017). It is documented that gene signatures and mechanistic molecular pathways were bridging the gap due to periodontitis and obesity, which were crucial in the transcriptomic analysis (Cai et al., 2023).

### 5.4 CVDs and neurodegenerative diseases

In CVDs such as myocardial infarction (MI), as well as stroke, immune responses are prominent as both destructive and restorative factors (Dounousi et al., 2021; Duni et al., 2024). The process of inflammation is viewed as a stage of tissue injury, but later immune processes associated with its regulation stimulate angiogenesis, scar tissue development, and restoration of lost functions (Muhammad et al., 2021; Rajput et al., 2025). Failure in the regulation of these processes could result in heart failure or chronic disability (Golino et al., 2024; Taylor et al., 2025). Similarly, neurodegenerative diseases (Huang et al., 2025), like Alzheimer’s disease (AD) (Gol Mohammad Pour Afrakoti et al., 2025; Liu et al., 2025) and diseases like Parkinson’s disease (PD), immune system failure results in neuronal loss and failure in the restoration of the neural architecture (Cai et al., 2023; Clark and Kodadek, 2016; Doroszkiewicz et al., 2024). These diseases are characterized by activation of microglia, chronic inflammation, and a lack of restorative ability (Golia et al., 2019; Z. Jiang et al., 2025). Modern recovery approaches advocate immunomodulation strategies, such as modulating neuroinflammation to optimize functional outcomes while alleviating disease progression (Lill et al., 2026; Molina-Holgado and Molina-Holgado, 2010; Morizane and Takahashi, 2021; Schwab et al., 2014). AD may result from alterations in the immune system, cell cycle, and protein processing due to changes in ribosome activity (Yamakawa et al., 2025).

Research indicates that changes in the adaptive immune system may fuel clinical disease in PD by shifting to a more proinflammatory state in the initiation of the PD patient cohort and points to possible immunotherapeutics targeting overactive CD4+ T, CD8+ T, and CD19+ B cell populations in PD patients (Elyaman, 2025; Roodveldt et al., 2024; Yan et al., 2021). The advancements in the field of both active and passive immunotherapies are of particular importance for future work detailing the role inflammation and immunity play in this disease (Du et al., 2025; Outeiro and El-Agnaf, 2023; Tan et al., 2020; Vedam-Mai, 2021). It has been shown that PD and MS have a pattern of associations that includes a decreased risk of lung cancer and an enhanced threat of breast cancer than would be anticipated (Ajdacic-Gross et al., 2016).

## 6 Determinants of recovery-immune interactions

### 6.1 Genetic and epigenetic effects

Immune activity and the potential for recovery of any tissue are mediated by several genetic factors. For example, predisposing infections, autoimmune activity, tissue repair associated with the various immunological phenotypes of HLA alleles (human leukocyte antigen alleles) and cytokine polymorphisms can influence immune responses and recovery in different ways (Mancuso et al., 2022; Rozevska et al., 2025; Wu et al., 2022). Other gene expression controlling factors like immune and regenerative pathway interacting DNA methylation, histones and non-coding RNAs are also considered epigenetic and are also highly determinant (Jia et al., 2024; Moorlag et al., 2024). Individual differences in recovery and therapy responses can be explained using the aforementioned molecular factors (Zhang and Cao, 2019). Recovery of immune function in acute and chronic disease infections relies on strategies informed by progress in epigenomics and immune system genomics (Burgess, 2015; Kou et al., 2023).

### 6.2 Nutritional and lifestyle factors

Nutrition and dietary practices are essential components of the recovery-immunity axis (Arzola-Martínez et al., 2023; Huo et al., 2025). Support of immune function and tissue repair particularly depends on the adequacy of the five main pillars of nutrition and micronutrients, especially vitamins A and C, and D, E, Zn, and the ω-3 fatty acids (Collins et al., 2024; Malek Mahdavi, 2020; Nieman and Mitmesser, 2017; Velleuer and Carlberg, 2020). The recovery processes, alongside extended phases of malnutrition or obesity, are especially hampered. Devastating lifestyle choices such as sedentarism, inadequate amounts of sleep, and use of other damaging substances like cigarettes and alcohol greatly diminish immunity and overall recovery potential (Grievink et al., 2022). Active rest facilitates more tissue repair and immune surveillance, and sleeping better augments the preparedness of the immunologic memory activation (Jesus et al., 2021; Mennitti et al., 2022). For these reasons, lifestyle change has been primary to improving recovery and immune competence (X. Gao et al., 2025).

### 6.3 Age, gender, and comorbidities

From a clinical and demographic perspective, the interplay of immune functions with recovery processes warrants further consideration (Yang et al., 2025). Aging is associated with the phenomena of immunosenescence and inflammaging, which further exacerbate the recovery from infections and chronic diseases (Catorce et al., 2021; Grievink et al., 2022). The differences between males and females concerning hormonal and genetic mechanisms of control account for differences in immunity and recovery, where females have a more effective immune response, but recovery rates are lower due to autoimmunity (Wu and Jin, 2025). The incidence of diabetes, hypertension, chronic kidney disease and other comorbidities further sustains immune recovery, which is also mechanistically muted due to inflammation and organ dysfunction (Doroszkiewicz et al., 2024; Girard and Vandiedonck, 2022; Hu et al., 2024). These considerations need to be incorporated in planning recovery, optimizing interventions for patients with diverse characteristics (Yang et al., 2025).

### 6.4 Psychosocial stress and mental health

Psychosocial matters substantially modify the association between recovery and immune system functioning (Branchi et al., 2024). Untreated chronic and unshakable stress, sadness, and other forms of anxiety are associated with the dysfunctioning of the hypothalamic-pituitary- adrenal (HPA) system, resulting in a more than normal presence of cortisol as well as immunity to other infections (Chiappelli et al., 2007; Y. Jiang et al., 2025). Such alterations slow the healing of wounds, the responsiveness to vaccines, and the recovery time from illness (Lei, 2025). Psychological resilience, social support and mental health have the opposite effect as immune responses and recovery are enhanced (Navarro-Ledesma et al., 2024). There is a need for a psychosocial approach to health during the medical and rehabilitation period to formulate more effective approaches to the complex recovery-immunity relationship (Branchi et al., 2024; X. Gao et al., 2025). To this end, a multi-omics systems vaccinology resource has been proposed to build and validate computational frameworks of immunity (Shinde et al., 2024).

## 7 Therapeutic and clinical implications

### 7.1 Immunotherapies and recovery improvements

Immunotherapy is an innovative intervention targeting immune functions to improve recovery outcomes (Fang et al., 2025). In oncology, immune checkpoint inhibitors and CAR-T cell therapies, which augment immune system functions to eliminate neoplastic cells and improve recovery post-therapy, are illustrative of such strategies (Jiang et al., 2022; Li et al., 2025). In autoimmune disorders, cytocloic biologics, such as TNF-α inhibitors and blockers of IL-6, restore dysregulated immunological processes to promote tissue healing and recovery of function (Mancuso et al., 2022). Other investigational regenerative strategies, like those employing mesenchymal stem cells (MSCs), are aimed at immunomodulation to enhance healing through immune suppression and amplified MSC therapies (Che et al., 2025). Targeted and controlled engagement of the immune system, therefore, holds substantial prospects for enhanced recovery across a spectrum of diseases (Harrell et al., 2021; Michel et al., 2020). It is known that immune memory is critical for the survival of immunopathology, and the authors recommend further empirical investigation to expose immunopathology at first infections and subsequent enduring immune defense (Cressler and Adelman, 2024).

**Figure 7:**
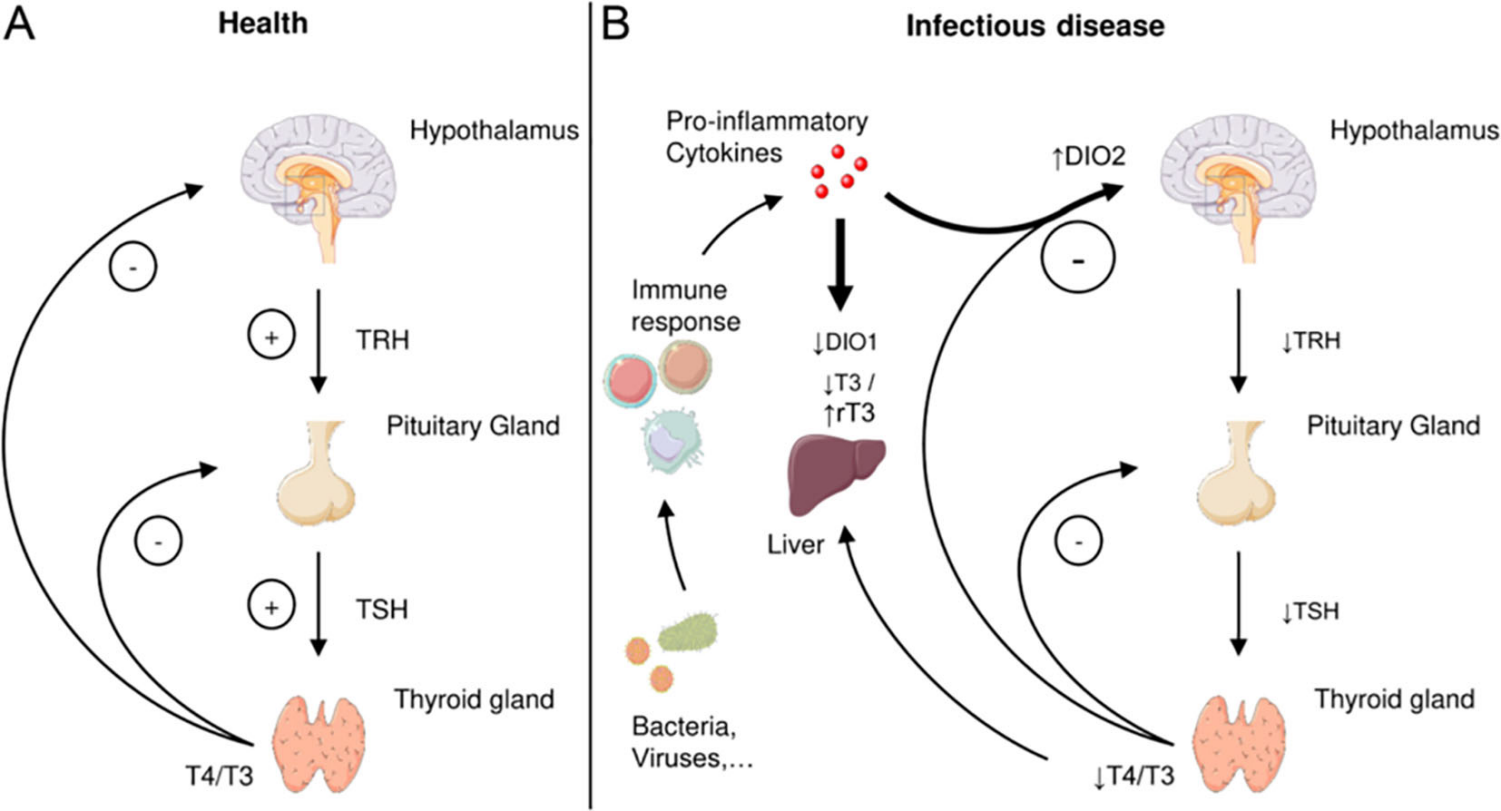
Health and Infectious disease. Biologists have concentrated on cross-primed coupled with systems biology to develop the systematic study of the HPT axis. **(A)** In the case of steady-state, the hypothalamus releases thyroliberin (TRH), which activates the pituitary gland to secrete thyroid-stimulating hormone (TSH). In response to T4, the hypothalamus and pituitary gland receive negative feedback. **(B)** In the case of infectious disease, proinflammatory cytokines in the periphery boost the negative feedback of thyroid hormones (TH) on the hypothalamus by increasing the DIO2 to decrease the secretion of TRH and TSH. Additionally, inflammatory signals alter the peripheral metabolism of thyroid hormones, leading to decreased DIO1 expression in the liver. Therefore, it is often observed that hypothyroidism occurs during severe illness, in which the serum concentration of T3 and/or T4, and in some cases, the concentration of reverse T3 (rT3) is elevated. Reprinted from Wenzek et al., 2022. Copyright © 2022, © The authors (Wenzek et al., 2022).

Unlike other viruses, oncolytic viruses (OVs) are unique in acting as viruses designed to lyse tumor cells, which work alongside the immune system (**Figure 7**). Almost all the work done, however, focuses on the role of mechanisms that work on the immune system (Liao and Watt, 2025). Consider the case of tumor clearance, OVs facilitate the clearance of the tumor and are designed with other therapeutic adjuncts to enhance clinical outcomes (DePeaux and Delgoffe, 2024). Regarding chronic gastric mucosal infections, several strategies targeting *Helicobacter pylori* suggest possible therapeutic targeting to evade the host’s innate and adaptive immune system. Global implementation of *H. pylori* vaccines will greatly enhance the chances of successfully eradicating such infections (Fan et al., 2024). In tauopathies, the phenomenon of tau aggregation and neuronal cell death is associated with extreme robust neuroinflammation, and especially certain types of microglial innate immune responses, which involve the central drivers of lipid metabolism, type I interferon signaling, and tau hyperphosphorylation and aggregation in relation to the adaptive immunity, together underlying the progression of tauopathies which have emerged with the participation of the immune system (Johnson and Lukens, 2025).

### 7.2 Rehabilitation and immune modulation

Rehabilitation involves, beyond repairing the body, immunological rehabilitation (Turk et al., 2024). Physical exercise can decrease systemic inflammation, enhance the immune response, and facilitate healing, especially in cardiovascular and metabolic disorders (Peake et al., 2017; Rajput et al., 2025). Among other things, neurorehabilitation techniques may lessen neuroinflammation, thereby aiding recovery in stroke and neurodegenerative disorders (Molina-Holgado and Molina-Holgado, 2010; Schwab et al., 2014; White et al., 2024). The use of AI has been reported for the prediction of independence in walking in stroke patients in the recovery-phase rehabilitation ward (Ono et al., 2024). More and more rehabilitation programs include cross-modally immune system influencing nutrition, psychology, and behavior (Huo et al., 2025; Nieman and Mitmesser, 2017). The rehabilitation outlined above, serving to resolve functional restrictions to immune system strength, recovery in infectious and non-communicable diseases, is an integrated, seamless approach (Senadheera et al., 2024; Wang et al., 2019). *Plasmodium falciparum*, in patients with chronic, asymptomatic malaria, remains more elusive. The transcriptions of the immune system’s var gene family are cloaked by reconnaissance of the antibodies that would usually surround it. It is known that parasites, once entered inside a cell, are present in a state with little or no var gene expression for decades. This alteration in var gene expression challenges the formerly held belief that var gene expression is reciprocally exclusive. Such immunological plasticity offers greater adaptive possibilities than previously understood (Florini et al., 2024).

### 7.3 Vaccination in recovery and immunity

Vaccination helps prevent disease and infection while also helping patients recover from disease by preparing a sophisticated response from the immune system (Batista et al., 2025; Walker et al., 2024). Vaccines for influenza, pneumococcus, and COVID- 19 lessen the burden of disease and its complications, which in turn leads to faster recovery (Ahmad et al., 2020; Augello et al., 2023; Cao et al., 2024; Ćwilichowska-Puślecka et al., 2025; Lauterio et al., 2020; Sridhar et al., 2015; Tiyo et al., 2021). In the context of cancer, therapeutic vaccines to enhance immune surveillance and prevent relapse are currently being investigated (Rios et al., 2025). In addition, vaccination of patients who are recovering from severe disease or those who are immunocompromised should be done cautiously in order to maximize beneficial effects while avoiding interference with ongoing recovery (Lee and Kim, 2025). Therefore, the rest of the researchers (Akbar et al., 2025; Augello et al., 2023; Chen et al., 2023) have shown that vaccination is still central to protective recovery strategies developed by multiple researchers.

The Lewy bodies formed in PD by the presynaptic protein alpha-synuclein (aSyn) and its abnormal excesses are another target whose therapeutic relevance lies in its association with different aspects of neuronal dysfunction and neurodegeneration, including neuroinflammation, defective mitochondria, disturbance in the protein degradatory pathways, and oxidative stress (Elyaman, 2025; Tansey and Romero-Ramos, 2019). Given the central role aSyn plays in neuroinflammation, aSyn-targeted immunotherapies are in the preclinical and clinical stages for PD (Du et al., 2025; Fleming et al., 2022). A live, attenuated *Yersinia pseudotuberculosis* plague vaccine that induces both innate and adaptive immunity has recently been reported and is claimed to be beneficial for the prevention and treatment of healthy and immunocompromised populations due to its enhanced safety and efficacy (Derbise et al., 2025).

Immune checkpoint inhibitors can result in patients with cancer achieving lasting disease control, but are limited to acute and chronic immune-related adverse events, which might necessitate immunosuppression and limit recovery (La-Beck and Owoso, 2024). Structured rehabilitation and, in particular, the pulmonary rehabilitation model has proven useful in the treatment of post-acute syndromes (e.g., long COVID), with protocols and effects that remain unstandardized and require personalization (Daynes et al., 2024; Zeraatkar et al., 2024). Vaccination is an effective preventive measure, as meta-analysis has shown that the risk of developing long COVID following infection is lower; however, this finding is not always consistent due to observational design and inter-study differences (Green et al., 2025). Lastly, endotype- and biomarker-based immunomodulation approaches for complex inflammatory conditions (e.g., sepsis) have focused on precision medicine to improve the alignment of therapy with immune status but have not yet been implemented due to the unavailability of assays, validation, and real-time clinical actionability (Giamarellos-Bourboulis et al., 2024).

### 7.4 Integrative and precision medicine approaches

Integrative and precision medicine approaches strive to customize rest and immunological interventions to distinct biopsychosocial characteristics of each patient (Kou et al., 2023). Precision immunotherapy translates through biomarker correlates (Sharma and Johnson, 2020; Zhou et al., 2024), genomic and clinical profiling (Khapuinamai et al., 2024), and tiered patient immune targeting (Arulkumaran et al., 2017). Integrative medicine includes nutrition, psychosocial stress management, and microbiome-centered strategies to augment immunity and recovery (Fidel Jr et al., 2020; Heidari et al., 2024; Swiatczak and Rescigno, 2012). The fusion of these approaches is useful to inform the design of ever-evolving, patient-specific strategies that enhance recovery while limiting negative consequences (Andres-Martin et al., 2024; Nieman and Mitmesser, 2017). Several fascinating proposals have been attributed to personalized vaccines able to bypass the issue of tumor heterogeneity, but the therapeutic gain is overshadowed by the inadequate antigen repertoire and deficient CD8+ T-cell responses. A simple yet effective way to obtain strong CD8+ T-cell immunization for personalized cancer immunotherapy involves bridging the gaps between innate and adaptive immunity (Kou et al., 2023). In addition, it has been pointed out that the restraint of the adaptive response to vaccines as part of precision medicine could be configured through the targeting of particular cell types, tissue sites, or receptors (Ke et al., 2022; Ung et al., 2023).

## 8 Future perspectives

### 8.1 Novel biomarkers of recovery and immunity

The use of important biomarkers is pivotal for the assessment of the ‘immunity recovery continuum’ (Arulkumaran et al., 2017; González-Pérez et al., 2025; Sarıdaş and Çetinkaya, 2025). The biomarkers being investigated for the ‘quality of recovery ‘ include circulating cytokine profiles, immune cell phenotypes, and indicators of tissue repair (González-Pérez et al., 2025; Rajput et al., 2025). Extracellular vesicles, metabolomic signatures and epigenetic changes confer molecular display between recovery and the immune system (Sarıdaş and Çetinkaya, 2025; Sharma and Johnson, 2020; Zhang and Cao, 2019). The performance of the procedure is correlated with the prediction on the backend ratio of still accurate diagnostics, customized therapeutic suggestions and insights on the prognosis for the long term (Arulkumaran et al., 2017; Licheng et al., 2024). It has been shown that PPP1R14B (protein phosphatase 1 regulatory subunit 14B) is a potential biomarker for the identification and prognosis of tumor immunity, proliferation and migration of immune and metastatic prostate cancer (Bao et al., 2024) and salivary lactoferrin as a biomarker for AD (Bermejo‐Pareja et al., 2020). Additionally, findings highlight a cross- cancer analysis that identifies CLEC5A (C-type lectin domain family 5 member A) as a prognostic biomarker for immunity across various cancers (Chen et al., 2022). It has recently been identified that GIMAP6 (GTPase of immunity-associated protein 6) acts as a prognostic biomarker for lung cancer tumor immunity (Chen et al., 2023) and HROB (homologous recombination factor with OB-fold) as a prognostic biomarker in renal cancer immunity (Ding et al., 2025). ABI3BP (ABI family member 3 binding protein) is a prognosis biomarker in lung tumor immunity infiltration (Feng et al., 2023), sortilin expression levels as a biomarker in PD patients (Georgoula et al., 2024), retinitis pigmentosa 2 (RP2) as a prognostic biomarker for glioma pathogenesis and tumor immune regulation (Gong et al., 2023), and MSH6 (MutS homolog 6) is a biomarker in the diagnosis and prognosis of bladder cancer with the immunity association (He et al., 2025). LGALS3BP (lectin galactoside-binding soluble 3 binding protein) is a potential prognostic biomarker in immunity linked with triple-negative breast cancer (A. Hu et al., 2025).

### 8.2 Systems biology and multi-omics approaches

Systems biology has developed a model to synthesize diverse data on recovery and immunity (Yu et al., 2019). Multi-omics approaches like genomics, transcriptomics, proteomics, metabolomics, and microbiomics expand the mapping of immune-recovery networks (Xu et al., 2024). Such methods uncover dependencies among molecular networks and facilitate the search for new therapeutic targets (Wen et al., 2025). Combined omics models that correlate with clinical outcomes may personalize approaches and enhance recovery trajectory predictive performance (Y. Gao et al., 2025; He and Wang, 2020). These methods have revealed predictive tumor immunity-associated molecular traits in the liver and colon cancers as features for enhancing anti-tumor immune response and response to immunotherapy (Elsayed et al., 2022; Hsieh et al., 2025; Shen et al., 2022). Recently, a study has focused on the lung cancer triad of inflammation, immunity, and metabolism, employing multi-omics to highlight interaction nodes with key inflammatory proteins such as N-acetyl-aspartyl-glutamate (NAAG). It articulated the roles of inflammation, the immune system, and metabolism in the pathogenesis and development of lung cancer, suggesting avenues for cross-cutting research on its treatment and control (Zhang et al., 2025).

### 8.3 Artificial intelligence and predictive models

**Figure 8:**
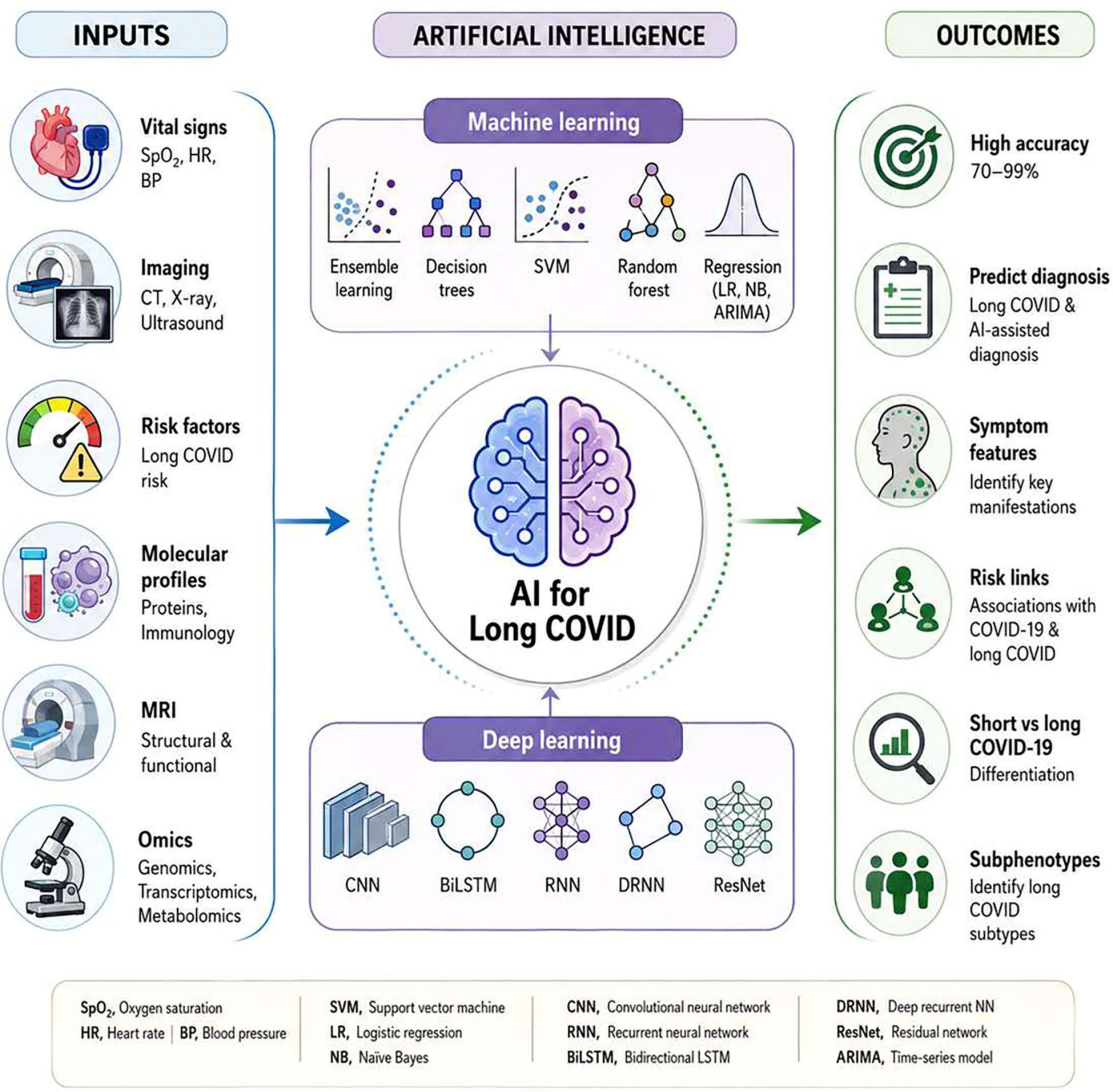
The proposed framework of AI applications in the early diagnosis of long COVID (Ashmawy et al., 2024).

It has been suggested that artificial intelligence (AI) and machine learning (ML) technologies have potential in analyzing datasets and forecasting recovery outcomes using markers of an individual’s immune response (Li et al., 2024). Predictive models can suggest patients likely to recover poorly and facilitate recovery planning with tailored individual clinical recovery pathways and recovery resource planning (Y. Gao et al., 2025). AI/ML models further improve drug discovery and therapy refinement by revealing new relationships of the immune system to recovery (Chao et al., 2025). The tools make it possible to convert recovery monitoring into a science of data predictive modeling and to perform multi-omics AI profiling of sepsis immunity (Y. Gao et al., 2025). Numerous studies have been conducted employing AI in the diagnostic process pertaining to long COVID (Ashmawy et al., 2024), breast cancer (Song et al., 2025), and even pancreatic cancer (Sidiropoulos et al., 2024). Utilizing the ML approach, key persistent immune response-modifying and secondary immune activation genes in responses to COVID-l9 recovery after vaccination with influenza have been identified (**Figure 8**) (Ren et al., 2024).

A recent study developed an AI-supported hybrid rehabilitation machine interfaced with a dual-arm robotic platform with suction-assisted EMG neuromuscular stimulation (NMES) to aid post-stroke upper limb recovery (White et al., 2024). The model achieved 95% accuracy in fatigue classification and dynamically responsive therapy, which tuned stimulation parameters according to fatigue classification results (Ben Abdallah et al., 2025). In new ventures within mental health, it has been reported that the ability of mental health LLMs to evaluate the prognosis of schizophrenia and achieved promising results (Elyoseph and Levkovich, 2024) and recovery from substance abuse has also been documented (Ramadhani et al., 2024).

### 8.4 Research gaps and opportunities

Much more work needs to be done in understanding the interfacing immunological factors and recovery. Outcome variability within and between populations (Georgoula et al., 2024; González-Pérez et al., 2025; Zuazo et al., 2020), psychosocial (Adriani et al., 2018; Sarıdaş and Çetinkaya, 2025), and biological determinants reflect voids needing integrated models. Span of cohort lessons (Sarıdaş and Çetinkaya, 2025) and creating new therapeutics (X. Wang et al., 2025; Zhou et al., 2024) alongside the cross-disease frameworks of communicable and non- communicable syndromes (Covache-Busuioc et al., 2025) are all within reach of more imaginative exploratory work. The global recovery and therapeutics as a whole need an opportunistic approach that fills these gaps for better recovery results.

There are significant gaps in the delineation of how immune programs mediate recovery from infectious and non-communicable diseases. The proposed future research should utilize recovery-specific immune biomarkers, supported by combined multi-omics profiling, to elucidate systemic and tissue-level recovery pathways (Giamarellos-Bourboulis et al., 2024). AI/ML-based models can also be used to stratify the risk of immune resilience, relapse, and chronic post-recovery, but must be validated in large longitudinal cohorts of long COVID, cancer survivorship, autoimmunity, and metabolic disease (Zeraatkar et al., 2024). Lastly, full control over immunomodulation to restore the balance of the immune system and leave host defense intact will be needed in order to put recovery as an active, quantifiable immunological condition into place (Saavedra-Torres et al., 2025).

## 9 Conclusion

The balance of recovery and immunity sinks health between pathology with tissue injury and exuberance. The hypothesis of not fully passive recovery and non-communicable inflammatory control illuminates the activity of the immune system. It is also evident that recovery dynamics of an immune system profoundly alter future immune recovery or competence thresholds with respect to new health issues. Exploration of immune system dysregulations amidst viral, bacterial, parasite, and fungal infections and cancer, and within autoimmune, metabolic, cardiovascular, and neurodegenerative conditions, elucidates the frameworks common to disparate pathologies. Nutrition, age, psychosocial factors, and genetics act to amplify this nexus, which intensifies the demand for personalized and integrative clinical governance. Immunotherapy, rehabilitation, precision medicine, and vaccination stand to strategically merge immune recovery with engagement along the optimal trajectory. The future clinical adoption of novel knowledge will derive from the identification of reliable biomarkers along with systems biology and the convergence of AI. Development of coherent strategies aimed at improving clinical outcomes demands bridging the gaps in the mechanisms of immune modulation and functional recovery. A broad, integrative perspective on the relationship between recovery and immunity suggests a fundamental shift in medicine. Rather than focusing on discrete disease entities, the emphasis should be on the intersection of immune pathways devoted to defensive, restorative, and resilient enduring health.
